# A multidimensional investigation of myelosuppression associated with sintilimab: integrating pharmacovigilance signal mining with real-world clinical evidence

**DOI:** 10.3389/fphar.2026.1784033

**Published:** 2026-04-10

**Authors:** Shiquan Liu, Shuying Liu, Yingwei Chang, Mingyang Nie, Yong Feng, Le Zhang

**Affiliations:** 1 Department of Thoracic Surgery, The Affiliated Hospital of Chengde Medical University, Chengde, China; 2 Radiology Department, South Hospital, Affiliated Hospital of Chengde Medical University, Chengde, China; 3 Department of Hand and Foot Surgery, South Hospital, Affiliated Hospital of Chengde Medical University, Chengde, China; 4 Department of Thoracic Surgery, The Fourth Affiliated Hospital of Hebei Medical University, Shijiazhuang, China

**Keywords:** immune checkpoint inhibitors, myelosuppression, pharmacovigilance, real-world study, sintilimab

## Abstract

**Objective:**

Sintilimab, a programmed cell death protein 1 (PD-1) inhibitor, is widely used in cancer immunotherapy, but its hematological toxicity profile in real-world settings remains incompletely understood. This study aims to comprehensively investigate the risk and characteristics of sintilimab-induced myelosuppression through integrated pharmacovigilance and clinical cohort analyses.

**Methods:**

We analyzed FDA Adverse Event Reporting System data (Q1 2020–Q2 2025) for sintilimab-associated adverse events using disproportionality analyses (Reporting Odds Ratio, Proportional Reporting Ratio). Time-to-onset was modeled using Weibull distribution, and risk factors were identified *via* LASSO and multivariable logistic regression. A retrospective cohort of 170 patients from a single center was analyzed to validate incidence, severity (CTCAE v5.0), and clinical predictors.

**Results:**

Among 668 FAERS reports, myelosuppression was the most frequent hematological adverse event (n = 146) with a strong signal (ROR = 51.86). In the clinical cohort, 67.06% developed myelosuppression, with 86.47% classified as Grade III/IV. Median onset was 14 days (IQR 5–30), with highest risk early in treatment. Independent risk factors included female sex (OR = 0.457, p = 0.013), paclitaxel use (OR = 4.129, p = 0.004), cisplatin use (OR = 2.240, p = 0.020), and advanced M stage (OR = 0.871, p = 0.006).

**Conclusion:**

Myelosuppression is a common, early-onset, and severe adverse event associated with sintilimab, particularly in patients receiving concomitant chemotherapy or with advanced disease. These findings underscore the need for intensified hematologic monitoring and personalized risk stratification in clinical practice.

## 1 Introduction

Immune checkpoint inhibitors (ICIs) that target the programmed cell death protein 1/programmed death-ligand 1 (PD-1/PD-L1) axis have significantly transformed therapeutic strategies for advanced malignancies (Zhang D. et al., 2024; Vickram et al., 2024; Zhou et al., 2025). Among these, sintilimab—a fully human IgG4 monoclonal antibody targeting PD-1—has demonstrated substantial efficacy and has been approved in China for multiple cancer types, including classical Hodgkin lymphoma, non-small cell lung cancer, and esophageal squamous cell carcinoma (Wu and Shao, 2025; Li et al., 2024).

The therapeutic efficacy of ICIs is associated with a unique range of immune-related adverse events (irAEs) that impact various organ systems (Wan et al., 2024; Sutanto et al., 2024). While dermatological, gastrointestinal, and endocrine toxicities are well-documented, hematological irAEs, especially myelosuppression, present a considerable clinical challenge (Fukushima et al., 2024; Han et al., 2025; Choi and Lee, 2020). This is due to their potential severity, their association with life-threatening complications, and their ability to interrupt the continuity of treatment.

The current understanding of the hematological toxicity associated with sintilimab is largely based on data from pivotal clinical trials. However, these trials may not accurately represent the real-world epidemiology, severity spectrum, and predisposing factors due to their selective patient populations and protocol-driven management approaches (Qin et al., 2024; Niblock et al., 2023). Spontaneous reporting databases, such as the FDA Adverse Event Reporting System (FAERS), serve as valuable resources for identifying post-marketing safety signals (Zhao et al., 2024). In contrast, well-characterized clinical cohorts provide detailed data on incidence and contextual risk factors (Lin et al., 2025).

To address these evidence gaps, the current study utilizes a convergent analytical approach comprising: (i) an extensive pharmacovigilance assessment of FAERS data to detect and characterize safety signals related to sintilimab, with a particular emphasis on hematological events; and (ii) a retrospective clinical cohort analysis to quantify the real-world impact and identify clinical determinants of myelosuppression. The integration of evidence from these complementary sources seeks to provide a more comprehensive and clinically relevant understanding of the myelosuppressive risks associated with sintilimab therapy.

## 2 Materials and methods

### 2.1 Collection and handling of pharmacovigilance information

In this study, FAERS data were used for signal detection and hypothesis generation, not for estimating the incidence or confirming causality of adverse events (Spadotto et al., 2025; Liu et al., 2025). We acknowledge the inherent limitations of spontaneous reporting databases, including reporting bias, incomplete data, and the lack of an exposed denominator, which preclude causal inference and incidence calculation. Reports submitted between the first quarter of 2020 and the second quarter of 2025 were extracted. This specific time frame was selected because the earliest adverse event reports involving sintilimab as the primary suspect drug in the FAERS database date back to Q1 2020, and the most recent complete data available at the time of analysis were through Q2 2025. Thus, this interval encompasses the entire spectrum of sintilimab-related reports from the first appearance to the latest update, ensuring a comprehensive and up-to-date dataset for pharmacovigilance assessment.

#### 2.1.1 Data source and initial extraction

The raw FAERS ASCII data files for the period Q1 2020 to Q2 2025 were downloaded from the FDA website. The initial download included three primary data tables: demographic information (DEMO, n = 9,197,318), drug information (DRUG, n = 42,834,727), and adverse reaction information (REAC, n = 23, 945, 657).

#### 2.1.2 Data cleaning and report selection

All data processing and statistical analyses were performed using R software (version 4.4.1) with the tidyverse and dplyr packages for data manipulation. The cleaning procedure followed a systematic multi-step algorithm.

##### 2.1.2.1 Duplicate removal

Within the DEMO table, duplicate records were identified based on CASEID, FDA_DT, and PRIMARYID. When multiple records shared the same CASEID, the record with the most recent FDA_DT and the highest PRIMARYID was retained. This process removed 1,372,042 duplicate records, resulting in a deduplicated DEMO table of 7,825,276 unique reports, as recommended in previous pharmacovigilance studies (Byskov et al., 2024).

##### 2.1.2.2 Identification of sintilimab reports

From the deduplicated DRUG table, we extracted all records where the drug name matched “sintilimab” using a standardized search dictionary. Only those reports in which sintilimab was listed as the primary suspect (PS) (ROLE_COD = “PS”) were retained. Reports with role codes “C” (concomitant) or “I” (interacting) were excluded. This step yielded 2,853 adverse event reports where sintilimab was identified as the PS.

##### 2.1.2.3 Linking to adverse event terms

The 2,853 reports were then linked to the REAC table to extract all preferred terms (PTs) associated with sintilimab as the PS. After removing reports with missing essential information (e.g., no valid outcome, incomplete demographic data) or those explicitly attributed to disease progression or non-drug causes, a final dataset of 668 reports was confirmed as drug-related adverse events unequivocally associated with sintilimab therapy.

##### 2.1.2.4 Final dataset

From these 668 reports, we extracted patient demographic characteristics (e.g., sex, age), clinical indications, outcome events, and time-to-onset information for subsequent pharmacovigilance analysis.

**FIGURE 1:**
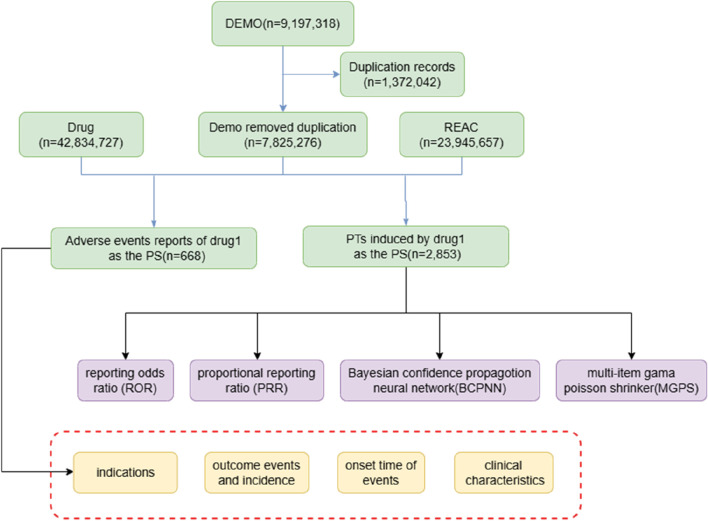
Diagram for detecting Sintilimab side effects in the FDA Adverse Event Reporting System (FAERS) database.

Figure 1 illustrates the complete data selection and analysis flow.

### 2.2 Identifying signals through disproportionality analysis

To detect potential safety signals associated with sintilimab, we conducted disproportionality analyses using four established algorithms: two frequentist methods (Reporting Odds Ratio (ROR) and Proportional Reporting Ratio (PRR)) and two Bayesian methods (Bayesian Confidence Propagation Neural Network (BCPNN) and Empirical Bayes Geometric Mean (EBGM)). All analyses were performed at both the System Organ Class (SOC) and PT levels (Zhang et al., 2026), in accordance with the Medical Dictionary for Regulatory Activities (MedDRA, version 28.1). The algorithms and their signal detection criteria are detailed below, with summary information provided in Supplementary Tables S1 and S2.

The ROR is a frequentist method that compares the odds of a specific adverse event (AE) for sintilimab against the odds of the same AE for all other drugs in the database. A signal was considered positive if the lower limit of the 95% confidence interval (CI) for the ROR was greater than 1, and the number of reported cases was at least 3 (Xia C. et al., 2024).

The PRR is another frequentist approach that compares the proportion of a specific AE for sintilimab to the proportion of the same AE for all other drugs. The criteria for a positive signal were: a PRR greater than or equal to 2, an associated chi-square (χ^2^) statistic of at least 4, and a minimum of three reported cases.

The BCPNN was used to measure the information component (IC), which represents the logarithm of the ratio of the observed to the expected reporting probability. A positive signal was defined when the lower bound of the 95% CI for the IC was greater than 0 (i.e., IC - 2 Standard Deviations SD > 0).

The EBGM was employed to calculate the geometric mean of the adjusted reporting ratio, providing a more stable estimate for rare events. A signal was deemed positive when the lower bound of the 95% CI for the EBGM exceeded 2.

These combined thresholds are consistent with established pharmacovigilance practices and are designed to minimize the identification of false-positive findings. In this study, a ‘positive safety signal’ is defined as an adverse event that met the signal detection criteria (i.e., ROR 95% CI lower bound >1, PRR ≥ 2 with χ^2^ ≥ 4, IC – 2SD > 0 or EBGM05 > 2) in any of the applied disproportionality analyses, indicating a statistically significant association with sintilimab.

### 2.3 Analysis of time-to-onset (TTO)

Time-to-onset (TTO) was defined as the interval between the start of sintilimab therapy and the occurrence of the adverse event. Only reports with complete and logically consistent dates were included. We acknowledge that TTO estimates from spontaneous reporting databases may be influenced by unrecorded clinical events such as disease progression, and therefore should be interpreted as descriptive rather than causal. The median and interquartile range (IQR) were used to describe central tendency and dispersion. A Weibull distribution was fitted to the TTO data using maximum likelihood estimation. The shape parameter (β) and its 95% confidence interval were estimated to characterize the hazard function over time. The hazard pattern is determined by whether the confidence interval includes 1: if the entire 95% CI is below 1, the hazard decreases over time; if it contains 1, the hazard is constant; if the entire CI is above 1, the hazard increases (Zhang W. et al., 2024; Sauzet and Cornelius, 2022).

### 2.4 Determining risk factors for myelosuppression (FAERS data)

A case–non-case design was applied. Cases were defined strictly as reports containing the PT “Myelosuppression” (PT code: 10028584) according to the Medical Dictionary for Regulatory Activities (MedDRA, version 28.1). Reports containing other cytopenia-related PTs (e.g., neutropenia, thrombocytopenia, anemia, leukopenia) were not classified as myelosuppression cases for this specific analysis, ensuring a focused and unambiguous evaluation of myelosuppression events associated with sintilimab. Non-cases comprised all other reports. Univariate logistic regression was first performed to screen potential predictors, including demographics, cancer type, and concomitant medications. Because a single FAERS report may list multiple cancer types as indications, each cancer type was treated as a separate, non-mutually exclusive binary variable to accurately reflect real-world clinical scenarios. Variables with a p-value <0.10 were entered into a Least Absolute Shrinkage and Selection Operator (LASSO) regression model to select the most parsimonious set of predictors while avoiding overfitting. The optimal penalty parameter (λ) was determined *via* 10-fold cross-validation using the one-standard-error rule. Variables with non-zero coefficients in the LASSO model were then included in a multivariable logistic regression model to estimate adjusted ORs and 95% CIs. For categorical variables, the reference categories were defined as follows: sex (reference: male), gastric cancer (reference: no), lung cancer (reference: no), esophageal cancer (reference: no), oxaliplatin (reference: no), paclitaxel (reference: no), and fluorouracil (reference: no). All statistical analyses were performed using R software (version 4.2.1), with the glmnet package used for LASSO regression. Missing data were handled by complete-case analysis due to the low proportion of missingness in key variables (<5%) (Hong et al., 2024). Cancer type was entered as a series of binary indicators as described above, with no single reference category, to capture the effect of each individual diagnosis.

### 2.5 Clinical group: research methodology and criteria for patient inclusion

A retrospective cohort study received approval from the Institutional Review Board of (Ethics Committee of the affiliated hospital of Chengde Medical University, blinded, No. CYFYLL2024669). The study included consecutive adult patients (aged 18 years and older) with histologically confirmed cancer who underwent at least one cycle of sintilimab treatment, either as monotherapy or in combination, between January 2024 and October 2025. Patients were excluded if they had pre-existing hematologic disorders, active severe infections, concurrent radiation therapy to major marrow-bearing sites, or insufficient clinical data.

### 2.6 Clinical cohort: analysis of outcomes and statistical evaluation

In this study, patients who had received Sintilimab without prior exposure to combination therapies were enrolled and subsequently categorized into two groups based on the presence or absence of myelosuppression following treatment: the non-myelosuppression group (n = 56) and the myelosuppression group (n = 114). Baseline clinical data were systematically collected, encompassing three categories of variables: demographic characteristics (including sex, classified as male or female, and age, presented as median and interquartile range [Q1, Q3]), tumor-related features (comprising tumor T stage [T1, T2, T3, T4, unknown], N stage [N0, N1, N2, N3, unknown], M stage [M0, M1, unknown], clinical stage [Stage I, II, III, IV], and cancer type [lung cancer, esophageal cancer]), and medication history (documented as the usage of Carboplatin, Nedaplatin, Cisplatin, and Bevacizumab, recorded as yes or no). For statistical analysis, the chi-square test was employed to compare categorical variables between the groups, while the Wilcoxon rank-sum test was utilized for continuous variables (age). P-values were calculated, with the significance level established at α = 0.05. A LASSO logistic regression analysis was conducted for the purpose of variable selection. The optimal Lambda value was identified by plotting the curve of binomial deviance against the logarithm of Lambda (log-transformed penalty coefficient), as well as examining the variable coefficient curve to identify potential influential variables associated with myelosuppression. Variables deemed statistically significant through the LASSO regression were subsequently incorporated into a multivariate logistic regression model to compute the odds ratio (OR) and 95% confidence interval (95% CI). For categorical variables, the reference categories were defined as follows: M stage (reference: M0) and Cisplatin usage (reference: no). This approach aimed to identify independent factors influencing myelosuppression, with statistical significance determined at a P-value threshold of <0.05. Additionally, for patients who developed myelosuppression, detailed information on clinical management was extracted from electronic medical records, including the use of supportive medications (granulocyte colony-stimulating factor [G-CSF], thrombopoietin [TPO] receptor agonists [e.g., eltrombopag], erythropoietin, blood transfusions), modifications to sintilimab or chemotherapy dosing (delays, reductions, or discontinuation), and the need for hospitalization or intensive care unit admission due to myelosuppression-related complications. Management decisions were based on standardized protocols aligned with international guidelines (CSCO, NCCN, ESMO, SITC). Key thresholds included: for neutropenia, G-CSF (5 μg/kg/day subcutaneously) was initiated when absolute neutrophil count (ANC) fell below 1.0 × 10^9^/L for treatment, or prophylactically in high-risk patients when ANC <1.5 × 10^9^/L; for thrombocytopenia, TPO receptor agonists were considered when platelet count was <50 × 10^9^/L, with platelet transfusion for counts <20 × 10^9^/L or active bleeding; for anemia, red blood cell transfusion was given for hemoglobin <7 g/dL or symptomatic anemia. Permanent discontinuation of sintilimab was implemented for grade ≥3 immune-related myelosuppression per guideline recommendations. Hospitalization was mandated for febrile neutropenia, defined as ANC <0.5 × 10^9^/L with a single oral temperature ≥38.3 °C or ≥38.0 °C sustained for 1 h.

## 3 Results

### 3.1 Sintilimab-related adverse event characteristics

Table 1 presents the clinical and demographic profile of AEs reports associated with Sintilimab. The analysis included 668 individual reports. A clear temporal increase was observed, with the number of reports rising from 14 (2.10%) in 2020 to a peak of 304 (45.51%) in 2024, followed by 86 (12.87%) in the partial year 2025.

**TABLE 1:** Sintilimab-related adverse event characteristics.

Variable	Total
Year	
2020	14 (2.10)
2021	48 (7.19)
2022	85 (12.72)
2023	131 (19.61)
2024	304 (45.51)
2025	86 (12.87)
Sex	
Female	181 (27.10)
Male	329 (49.25)
Unknown	158 (23.65)
Age, years	59.00 (50.00, 67.00)
<19	4 (0.62)
19–44	69 (10.68)
44–59	165 (25.54)
≥60	249 (38.54)
Unknown	159 (24.61)
Weight, kg	60.00 (53.00, 68.00)
<50	40 (5.99)
50–100	217 (32.49)
Unknown	411 (61.53)
Reporter	
Pharmacist	464 (69.46)
Physician	145 (21.71)
Consumer	50 (7.49)
Unknown	9 (1.35)
Indication	
Lung cancer	174 (19.40)
Hepatic cancer	167 (16.39)
Gastric cancer	97 (15.05)
Colorectal cancer	59 (6.58)
Squamous cell carcinoma	30 (3.34)
Esophageal cancer	27 (3.01)
Diffuse large B-cell lymphoma	24 (2.68)
Renal cancer	23 (2.56)
Invasive ductal carcinoma	23 (2.56)
Other	250 (27.87)
Unknown	5 (0.56)
Reported countries	
China	650 (97.31)
Germany	5 (0.75)
India	1 (0.15)
Netherlands	1 (0.15)
United States	1 (0.15)
Other	10 (1.50)
Route	
Intravenous drip	194 (27.17)
Intravenous	153 (21.43)
Other	367 (51.40)
Outcomes	
Hospitalization	228 (26.67)
Death	72 (8.42)
Life threatening	67 (7.84)
Disability	25 (2.92)
Congenital anomaly	1 (0.12)
Other serious	462 (54.04)
TTO, days	20.00 (5.00, 38.00)
<2	57 (12.47)
2–5	25 (5.47)
5–7	18 (3.94)
7–14	47 (10.28)
14–28	86 (18.82)
≥28	73 (15.97)
Unknown	151 (33.04)

The cohort comprised more males (329, 49.25%) than females (181, 27.10%), with sex unreported in 158 cases (23.65%). The median age was 59.0 years (interquartile range [IQR]: 50.0–67.0). The majority of patients (249, 38.54%) were aged 60 years or older, while a smaller proportion (4, 0.62%) were under 19 years. Median body weight was 60.0 kg (IQR: 53.0–68.0).

Regarding the indications for sintilimab use, the most frequently reported cancer types were lung cancer (174 reports, 19.40% of all indication entries), hepatic cancer (167 reports, 16.39%), and gastric cancer (97 reports, 15.05%). Other common indications included colorectal cancer (59 reports, 6.58%), squamous cell carcinoma (30 reports, 3.34%), esophageal cancer (27 reports, 3.01%), diffuse large B-cell lymphoma (24 reports, 2.68%), renal cancer (23 reports, 2.56%), and invasive ductal carcinoma (23 reports, 2.56%). A substantial proportion of reports listed other or unspecified cancer types (250 reports, 27.87%), and five reports (0.56%) had missing indication data.

Pharmacists submitted the majority of reports (464, 69.46%), followed by physicians (145, 21.71%). Geographically, reports were predominantly from China (650, 97.31%), with sporadic cases from Germany, India, the Netherlands, the United States, and other regions.

Regarding administration, intravenous drip (194, 27.17%) and intravenous injection (153, 21.43%) were documented, while a substantial proportion of reports (367, 51.40%) listed other or unspecified routes. The most frequently reported serious outcome category was “other serious” AEs (462, 54.04%). Hospitalization was required in 228 cases (26.67%), death was reported in 72 (8.42%), and life-threatening events occurred in 67 (7.84%). Less frequent serious outcomes included disability (25, 2.92%) and one case of congenital anomaly (0.12%).

These findings delineate the key characteristics of Sintilimab-related AE reports, highlighting trends over time, patient demographics, reporting patterns, and the spectrum of documented serious outcomes.

### 3.2 Adverse events associated with sintilimab and their system organ class characteristics

**FIGURE 2:**
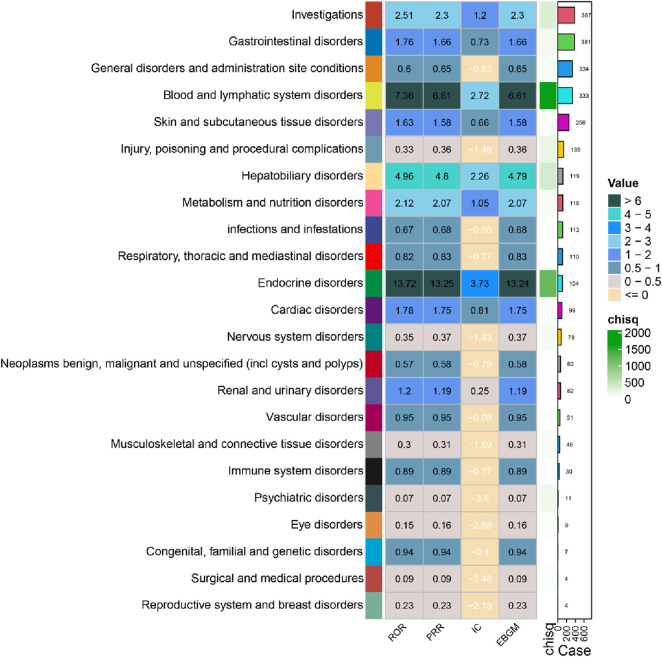
Adverse events associated with Sintilimab and their system organ class characteristics.

The distribution of AEs in FAERS database associated with Sintilimab across SOCs revealed distinct patterns of disproportionate reporting, as summarized in Figure 2. Among 23 SOCs analyzed, the most frequently reported AEs fell under “investigations” (387 cases), followed by “gastrointestinal disorders” (381 cases) and “general disorders and administration site conditions” (334 cases).

Notably, several SOCs demonstrated strong positive safety signals. The most prominent signal was observed for “endocrine disorders” (104 cases), with a Reporting Odds Ratio (ROR) of 13.72 (95% CI: 11.28–16.69) and a Proportional Reporting Ratio (PRR) of 13.25 (95% CI: 10.89–16.12). Similarly, “blood and lymphatic system disorders” (333 cases) and “hepatobiliary disorders” (119 cases) showed significant positive associations, with RORs of 7.36 (95% CI: 6.56–8.25) and 4.96 (95% CI: 4.13–5.96), respectively. Other SOCs with elevated signals included “metabolism and nutrition disorders” (ROR = 2.12) and “cardiac disorders” (ROR = 1.78).

Conversely, several SOCs exhibited significant negative signals, suggesting a lower-than-expected reporting association with Sintilimab. These included “psychiatric disorders” (ROR = 0.07), “eye disorders” (ROR = 0.15), “musculoskeletal and connective tissue disorders” (ROR = 0.30), “injury, poisoning and procedural complications” (ROR = 0.33), and “nervous system disorders” (ROR = 0.35).

Collectively, these data indicate that Sintilimab-associated AEs are predominantly concentrated within specific organ systems, particularly endocrine, hematologic, and hepatobiliary systems, while showing reduced association with psychiatric, neurological, and musculoskeletal disorders.

### 3.3 Adverse events associated with sintilimab and their preferred terms characteristics

**FIGURE 3:**
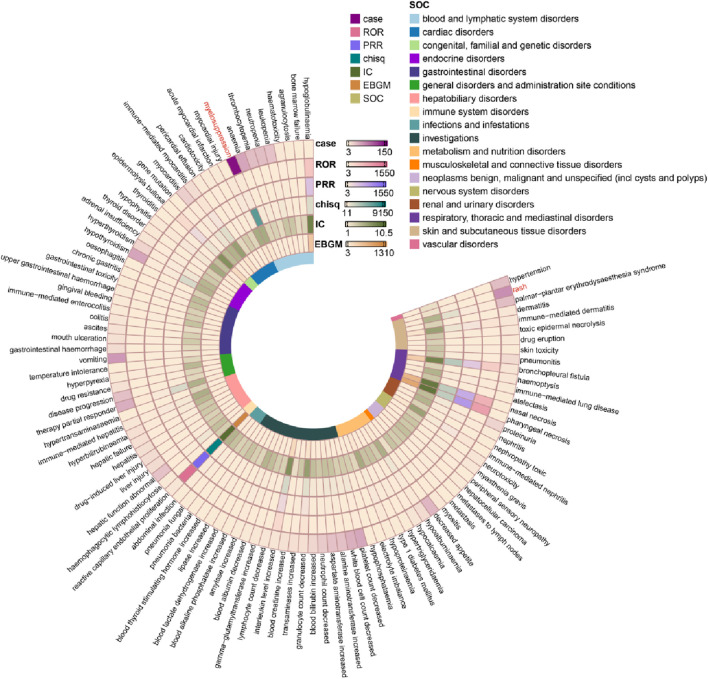
Adverse events associated with Sintilimab and their PTs characteristics.

An in-depth analysis at the PT level further delineated the specific AEs most strongly associated with Sintilimab, as detailed in Figure 3. Among the most frequently reported PTs were myelosuppression (146 cases, ROR 51.86, 95% CI: 43.88–61.28), rash (67 cases, ROR 3.26), vomiting (60 cases, ROR 3.16), and hypothyroidism (51 cases, ROR 32.87). Notably, several PTs demonstrated exceptionally high disproportionality signals, indicative of a strong safety concern. These included immune-mediated myocarditis (ROR 94.15), bronchopleural fistula (ROR 391.43), and most strikingly, reactive capillary endothelial proliferation (ROR 1549.72) (Supplementary Table S3).

The spectrum of high-signal PTs was distributed across key SOCs. Prominent signals within blood and lymphatic system disorders comprised myelosuppression, anaemia (ROR 6.68), thrombocytopenia (ROR 7.08), and leukopenia (ROR 14.24). For endocrine disorders, in addition to hypothyroidism, strong signals were observed for hyperthyroidism (ROR 27.73) and hypophysitis (ROR 56.06). Hepatobiliary disorders were characterized by PTs such as hepatic function abnormal (ROR 18.21), drug-induced liver injury (ROR 7.07), and hepatitis (ROR 11.21). Furthermore, immune-mediated events featured prominently, encompassing immune-mediated dermatitis (ROR 91.16), immune-mediated hepatitis (ROR 42.46), and immune-mediated enterocolitis (ROR 15.87).

Additional notable PTs with elevated signals included platelet count decreased (ROR 9.87), palmar-plantar erythrodysaesthesia syndrome (ROR 31.98), myocarditis (ROR 32.92), proteinuria (ROR 20.64), and pneumonitis (ROR 10.68). These findings at the PT level substantiate and refine the SOC-level analysis, identifying a distinct profile of Sintilimab-associated AEs dominated by hematological toxicity, endocrine dysfunction, hepatobiliary injury, and a range of immune-related adverse events.

### 3.4 Time to onset

The time-to-onset analysis of all sintilimab-associated adverse events revealed a variable temporal profile. The distribution of onset intervals for the complete spectrum of reported AEs was as follows: events occurring within 2 days accounted for 12.47% (57 cases), 2–5 days for 5.47% (25 cases), 5–7 days for 3.94% (18 cases), 7–14 days for 10.28% (47 cases), 14–28 days for 18.82% (86 cases), and beyond 28 days for 15.97% (73 cases) (Table 1).

A Weibull distribution model was fitted to the onset data, yielding a shape parameter (β) of 0.785 (95% CI: 0.719–0.850). Because the upper limit of the 95% confidence interval (0.850) is less than 1, this indicates a statistically significant decreasing hazard over time. These findings suggest that the risk of sintilimab-related adverse events is highest during the early treatment period and gradually declines thereafter.

These findings characterize the onset kinetics of Sintilimab-associated AEs, supporting a pattern of early risk concentration followed by a reduced hazard in prolonged exposure.

### 3.5 Analysis of risk factors for myelosuppressions

To identify risk factors for myelosuppression using pharmacovigilance data, a retrospective case/non-case analysis was conducted on reports from the FAERS. This analysis compared baseline characteristics, clinical indications, and concomitant medications between patients who developed myelosuppression and those who did not within this global database of spontaneous reports. Multivariate logistic regression and least absolute shrinkage and selection operator (LASSO) regression were further applied to identify independent risk factors and refine variable selection.

This sub-analysis included 217 patients, of whom 76 developed myelosuppression and 141 did not. As shown in Table 2, age (median [Q1, Q3]: 59 [51, 68] vs. 63 [53, 70] years; P = 0.283) and body weight (mean ± SD: 59.78 ± 10.47 kg vs. 61.64 ± 10.63 kg; P = 0.216) did not differ significantly between groups. However, sex distribution varied markedly (P = 0.009): the myelosuppression group had a higher proportion of females (44.74% vs. 26.24%) and a lower proportion of males (55.26% vs. 73.76%).

**TABLE 2:** Comparison of baseline traits and medication exposure between patients with and without myelosuppression.

Variables	Total (n = 217)	Non-myelosuppression (n = 141)	Myelosuppression (n = 76)	P. value
Patient characteristics				
Age, median (Q1, Q3)	61 (52, 69)	63 (53, 70)	59 (51, 68	0.283
Sex, n (%)	​	​	​	0.009
Female	71 (32.72)	37 (26.24)	34 (44.74)	​
Male	146 (67.28)	104 (73.76)	42 (55.26)	​
Weight, mean ± SD	60.99 ± 10.59	61.64 ± 10.63	59.78 ± 10.47	0.216
Indication				
Gastric cancer, n (%)	​	​	​	0.052
No	156 (71.89)	108 (76.6)	48 (63.16)	​
Yes	61 (28.11)	33 (23.4)	28 (36.84)	​
Hepatic cancer, n (%)	​	​	​	0.681
No	196 (90.32)	126 (89.36)	70 (92.11)	​
Yes	21 (9.68)	15 (10.64)	6 (7.89)	​
Lung cancer, n (%)	​	​	​	0.112
No	198 (91.24)	125 (88.65)	73 (96.05)	​
Yes	19 (8.76)	16 (11.35)	3 (3.95)	​
Colon cancer, n (%)	​	​	​	0.751
No	206 (94.93)	133 (94.33)	73 (96.05)	​
Yes	11 (5.07)	8 (5.67)	3 (3.95)	​
Esophageal cancer, n (%)	​	​	​	0.061
No	216 (99.54)	133 (94.33)	73 (96.05)	​
Yes	11 (5.07)	8 (5.67)	3 (3.95)	​
Drug				
Oxaliplatin, n (%)	​	​	​	0.350
No	159 (73.27)	108 (76.6)	51 (67.11)	​
Yes	58 (26.73)	33 (23.4)	25 (32.89)	​
Paclitaxel, n (%)	​	​	​	0.006
No	193 (88.94)	132 (93.62)	61 (80.26)	​
Yes	24 (11.06)	9 (6.38)	15 (19.74)	​
Irinotecan, n (%)	​	​	​	0.660
No	212 (97.7)	137 (97.16)	75 (98.68)	​
Yes	5 (2.3)	4 (2.84)	1 (1.32)	​
Fluorouracil, n (%)	​	​	​	0.132
No	209 (96.31)	138 (97.87)	71 (93.42)	​
Yes	8 (3.69)	3 (2.13)	5 (6.58)	​
Carboplatin, n (%)	​	​	​	0.955
No	201 (92.63)	130 (92.2)	71 (93.42)	​
Yes	16 (7.37)	11 (7.8)	5 (6.58)	​
Cisplatin, n (%)	​	​	​	1.000
No	205 (94.47)	133 (94.33)	72 (94.74)	​
Yes	12 (5.53)	8 (5.67)	4 (5.26)	​

^a^ Because a single report in the FAERS, database may list multiple indications (cancer types), these categories are not mutually exclusive. Each cancer type was treated as a separate binary variable (yes/no) to accurately capture the clinical reality of patients with multiple malignancies. Percentages are calculated based on the total number of patients in each group. P-values are derived from chi-square or Fisher’s exact tests for categorical variables and the Wilcoxon rank-sum test for age.

With respect to tumor type, gastric cancer showed a borderline association with myelosuppression (36.84% vs. 23.40%; P = 0.052). A similar trend was observed for esophageal cancer (P = 0.061), whereas no significant differences were found for hepatic, lung, or colon cancer (all P > 0.05).

Among concurrent chemotherapeutic agents, paclitaxel exposure was significantly higher in the myelosuppression group (19.74% vs. 6.38%; P = 0.006). No significant differences were detected for oxaliplatin, irinotecan, fluorouracil, carboplatin, or cisplatin (Table 2).

**FIGURE 4:**
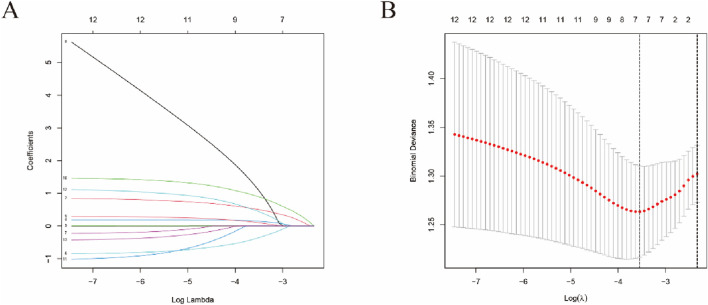
LASSO regression analysis for risk factor selection in the FAERS cohort. **(A)** Binomial deviance plot showing the cross-validation error as a function of log(λ). The left vertical dotted line indicates the λ value that minimizes the mean squared error (λ.min), while the right vertical dotted line represents the λ value selected using the one-standard-error rule (λ.1se), which was used for final model selection. **(B)** Coefficient path plot showing the shrinkage of regression coefficients for each candidate predictor as log(λ) increases. Each colored line represents a different clinical variable (sex, cancer types, and concomitant medications). As the penalty increases, coefficients shrink toward zero, with only the most important predictors (those with non-zero coefficients at the optimal λ) retained for subsequent multivariate logistic regression. Variables with non-zero coefficients at the selected λ included sex, gastric cancer, lung cancer, esophageal cancer, oxaliplatin, paclitaxel, and fluorouracil.

In the LASSO regression analysis, which appropriately handled cancer types as non-mutually exclusive binary variables, a set of potential predictors with non-zero coefficients was identified (Figures 4A,B). These potential risk factors included sex, gastric cancer, lung cancer, esophageal cancer, oxaliplatin, paclitaxel, and fluorouracil. Multivariate logistic regression (Table 3), with male sex as the reference category, confirmed female sex as an independent protective factor against myelosuppression (OR = 0.457, 95% CI: 0.245–0.847; P = 0.013), indicating that female patients had approximately 54% lower odds of reporting myelosuppression compared to males. Conversely, with no paclitaxel use as the reference, paclitaxel use emerged as the strongest independent risk factor (OR = 4.129, 95% CI: 1.605–11.260; P = 0.004), conferring a more than fourfold increased risk. Clinical indications and other chemotherapy agents did not retain independent significance.

**TABLE 3:** Logistic regression analysis using multiple variables.

Variables	Multivariate analysis OR (95% CI)	P. value
Patient characteristics		
Sex	0.457 (0.245–0.847)	0.013
Indication		
Gastric cancer	1.292 (0.615–2.692)	0.494
Lung cancer	0.401 (0.086–1.363)	0.182
Esophageal cancer	0.000 (0.000-inf)	0.986
Drug		
Oxaliplatin	1.332 (0.617–2.840)	0.459
Paclitaxel	4.129 (1.605–11.260)	0.004
Fluorouracil	2.751 (0.611–14.469)	0.194

### 3.6 Fundamental traits of the study group and the incidence of myelosuppression following sintelimab administration

To complement the pharmacovigilance findings with detailed clinical data, we next analyzed our institutional retrospective cohort of patients treated with sintilimab. Between January 2024 and October 2025, a total of 170 eligible patients from the Affiliated Hospital of Chengde Medical University were enrolled in the study. Comprehensive baseline clinical characteristics are detailed in Supplementary Table S3. The cohort consisted of four females (2.35%) and 166 males (97.65%), with a mean age of 63.51 years. The study included two cancer types, with esophageal cancer being the most prevalent (n = 119, 70.00%). The treatment regimens incorporated four combination agents: carboplatin, nedaplatin, cisplatin, and bevacizumab. Supplementary Table S4 illustrates the specific stage of the tumor and the medication combination. Among the 170 patients treated with sintilimab, 114 (67.06%) developed myelosuppression. According to CTCAE version 5.0, the severity distribution among these 114 patients was as follows: grade I in 69 patients (40.59%), grade II in 32 patients (18.82%), grade III in 7 patients (4.12%), and grade IV in 6 patients (3.53%) (Supplementary Table S5). Regarding clinical management of these events, interventions strictly followed international guideline recommendations:

Neutropenia: Of the 114 patients, 28 (24.56%) experienced neutropenia (ANC <2.0 × 10^9^/L). Among those with grade ≥3 neutropenia (ANC <1.0 × 10^9^/L, n = 8), all received G-CSF at 5 μg/kg/day subcutaneously until recovery to grade ≤1 (ANC ≥1.5 × 10^9^/L). The median duration of G-CSF therapy was 7 days (IQR 5–10 days). The median ANC at G-CSF initiation was 0.36 × 10^9^/L (IQR 0.24–0.65 × 10^9^/L).

Thrombocytopenia: Thrombocytopenia (platelet count <100 × 10^9^/L) occurred in 64 patients (56.14%). For grade ≥3 thrombocytopenia (platelet count <50 × 10^9^/L, n = 1), and platelet transfusions (platelet count <20 × 10^9^/L or active bleeding). No intracranial hemorrhages were observed.

Regarding anemia: Consistent with our data presented in Supplementary Table S5, no patients in our cohort developed anemia requiring intervention.

Regarding dose modifications: In our cohort, no dose reductions or delays of sintilimab or concomitant chemotherapy were implemented for myelosuppression management. All patients continued treatment as scheduled, with supportive care provided as needed.

Regarding hospitalization: All 114 patients (100%) who developed myelosuppression were hospitalized for management.

### 3.7 Evaluation of initial characteristics before starting sintilimab combination therapy

**FIGURE 5:**
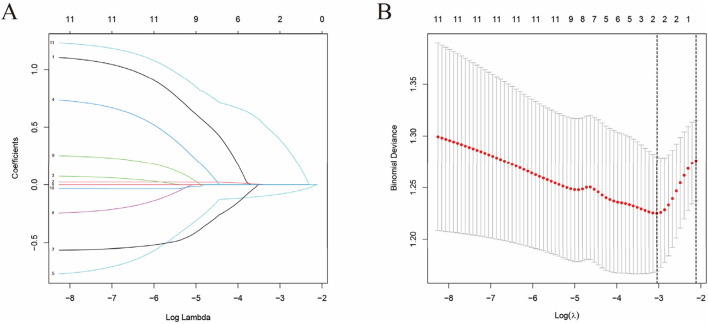
LASSO regression analysis for risk factor selection in the clinical cohort. **(A)** Binomial deviance curve plotted against log(λ). The y-axis represents binomial deviance (a measure of model fit), and the x-axis shows the log-transformed penalty parameter. The optimal λ value was selected *via* 10-fold cross-validation using the one-standard-error rule, corresponding to the most regularized model with cross-validation error within one standard error of the minimum. **(B)** Coefficient trajectory plot demonstrating the dynamic shrinkage of variable coefficients as the penalty parameter increases. Each curve represents a candidate predictor variable (including T stage, N stage, M stage, clinical stage, and concomitant medications). With increasing log(λ), coefficients progressively shrink toward zero, and variables that maintain non-zero coefficients at the optimal λ (M stage and Cisplatin use) were identified as potentially important predictors of myelosuppression and retained for multivariate logistic regression analysis. The colors correspond to different predictor variables as shown in the inset.

A comparison of baseline characteristics between the two groups, as presented in Table 4, indicated no statistically significant differences in the distribution of sex (P = 0.665), age (P = 0.192), presence of lung cancer (P = 0.342), presence of esophageal cancer (P = 0.342), usage of Carboplatin (P = 0.473), usage of Nedaplatin (P = 0.163), or usage of Bevacizumab (P = 1.0). Conversely, statistically significant differences were identified in tumor-related characteristics and medication history. Specifically, for tumor-related features, significant differences were found in T stage (P = 0.014), M stage (P = 0.003), and clinical stage (P = 0.028), with a higher proportion of patients in the myelosuppression group exhibiting T3 stage (48.25% vs. 25%), M0 stage (59.65% vs. 39.29%), and Stage III (40.35% vs. 21.43%). Regarding medication history, the usage rate of Cisplatin was significantly higher in the myelosuppression group (69.3%) compared to the non-myelosuppression group (46.43%), with this difference being statistically significant (P = 0.007). Moreover, the N stage exhibited a marginally statistically significant difference (P = 0.053), approaching the conventional significance threshold of α = 0.05. The proportions of patients at N1 and N2 stages were slightly elevated in the myelosuppression group. In the process of variable selection using LASSO logistic regression (Figure 5), the binomial deviance initially decreased and subsequently stabilized as the log-transformed penalty coefficient (log Lambda) increased, reaching its minimum at the optimal Lambda value. The variable coefficient trajectory demonstrated that the regression coefficients of certain variables progressively diminished to zero with increasing log Lambda. Ultimately, variables with non-zero coefficients, such as M stage and Cisplatin usage, were retained for further multivariate regression analysis. The findings from the multivariate logistic regression analysis (Table 5), with M0 as the reference category for M stage, indicate that M stage serves as an independent protective factor against myelosuppression (OR = 0.871, 95% CI: 0.788–0.960, P = 0.006). This suggests that, relative to M0, each incremental increase in M stage category is associated with a 12.9% reduction in the odds of myelosuppression. Conversely, with no Cisplatin use as the reference, the use of Cisplatin emerged as an independent risk factor for myelosuppression (OR = 2.240, 95% CI: 1.133–4.450, P = 0.020), indicating that patients receiving Cisplatin have 2.24 times higher odds of developing myelosuppression compared to those not receiving this treatment.

**TABLE 4:** A comparison of initial characteristics between patients who experienced myelosuppression and those who did not after receiving Sintilimab, before being exposed to combination drugs.

Variables	Non-myelosuppression (n = 56)	Myelosuppression (n = 114)	P. value
Patient characteristics			
Sex, n (%)	​	​	0.665
Male	55 (98.21)	109 (95.61)	​
Female	1 (1.79)	5 (4.39)	​
Age, median (Q1,Q3)	63 (57, 67)	64 (57, 69.5)	0.192
Stage			
T, n (%)	​	​	0.014
T1	2 (3.57)	6 (5.26)	​
T2	9 (16.07)	10 (8.77)	​
T3	14 (25)	55 (48.25)	​
T4	14 (25)	27 (23.68)	​
Unknown	17 (30.36)	16 (14.04)	​
N, n (%)	​	​	0.053
N0	4 (7.14)	6 (5.26)	​
N1	14 (25)	35 (30.7)	​
N2	12 (21.43)	35 (30.7)	​
N3	10 (17.86)	26 (22.81)	​
Unknown	16 (28.57)	12 (10.53)	​
M, n (%)	​	​	0.003
M0	22 (39.29)	68 (59.65)	​
M1	17 (30.36)	34 (29.82)	​
Unknown	17 (30.36)	12 (10.53)	​
Stage, n (%)	​	​	0.028
I	1 (1.79)	0 (0)	​
II	7 (12.5)	15 (13.16)	​
III	12 (21.43)	46 (40.35)	​
IV	36 (64.29)	53 (46.49)	​
Cancer			
Lung cancer, n (%)	​	​	0.342
No	35 (62.5)	81 (71.05)	​
Yes	21 (37.5)	33 (28.95)	​
Esophageal cancer, n (%)	​	​	0.342
No	21 (37.5)	33 (28.95)	​
Yes	35 (62.5)	81 (71.05)	​
Drug			
Carboplatin, n (%)	​	​	0.473
No	45 (80.36)	98 (85.96)	​
Yes	11 (19.64)	16 (14.04)	​
Nedaplatin, n (%)	​	​	0.163
No	45 (80.36)	102 (89.47)	​
Yes	11 (19.64)	12 (10.53)	​
Cisplatin, n (%)	​	​	0.007
No	30 (53.57)	35 (30.7)	​
Yes	26 (46.43)	79 (69.3)	​
Bevacizumab, n (%)	​	​	1
No	54 (96.43)	110 (96.49)	​
Yes	2 (3.57)	4 (3.51)	​

**TABLE 5:** Logistic regression analysis using multiple variables.

Variables	Multivariate analysis OR (95% CI)	P. value
Stage		
M	0.871 (0.788–0.960)	0.006
Drug		
Cisplatin	2.240 (1.133–4.450)	0.020

## 4 Discussion

ICIs targeting the PD-1/PD-L1 axis have revolutionized the treatment of advanced malignancies, yet their unique spectrum of irAEs poses significant clinical challenges (Huang et al., 2022; Xia Z. et al., 2024; Xu et al., 2023). While hematological toxicities such as myelosuppression are recognized complications, their real-world epidemiology and risk factors remain poorly characterized, largely due to the selective nature of clinical trial populations. This integrated study, combining pharmacovigilance signal mining from the FAERS database with a retrospective clinical cohort analysis, provides a comprehensive assessment of the safety profile of sintilimab, with a particular focus on myelosuppression. Our findings reveal a markedly high incidence (67.06%) and severity (86.47% Grade III/IV) of myelosuppression in real-world practice—substantially exceeding rates reported in pivotal trials—and confirm a strong statistical association with sintilimab exposure (ROR = 51.86) in the FAERS database. These results underscore that myelosuppression is not only a statistically detectable signal but also a clinically dominant and often severe toxicity in routine care, warranting prioritized monitoring and proactive management.

ICIs that target the PD-1/PD-L1 axis have significantly transformed the therapeutic landscape for advanced malignancies. Sintilimab, a fully human IgG4 anti-PD-1 monoclonal antibody, has been extensively integrated into clinical practice due to its demonstrated efficacy across a variety of tumor types. Nevertheless, the distinct spectrum of irAEs associated with ICIs continues to present challenges for their safe clinical application. Among these irAEs, hematological complications, particularly myelosuppression, have attracted increasing attention due to their potential severity and impact on treatment continuity. Despite this, the real-world epidemiology and risk factors associated with these adverse events remain inadequately characterized. This study, which integrates pharmacovigilance signal mining from the FAERS with a single-center retrospective clinical cohort analysis, offers comprehensive insights into the safety profile of sintilimab, with a particular emphasis on myelosuppression.

A significant finding of this study, derived from the real-world clinical cohort, is the high incidence of myelosuppression associated with sintilimab. Among 170 patients treated at our center, 67.06% experienced myelosuppression, with Grade III/IV severe events comprising 86.47% of cases—substantially surpassing the incidence rates reported in pivotal clinical trials. In contrast, the FAERS database was used not to estimate incidence, but to detect disproportionality signals and identify risk factors associated with myelosuppression, complementing the clinical findings with pharmacovigilance evidence. For instance, in the ORIENT-11 trial, the most common Grade ≥3 AEs in the sintilimab plus chemotherapy group were decreased neutrophil count (36.5%), anemia (15.0%) and decreased white blood count (14.7%). Specifically, Grade ≥3 neutropenia occurred in 26.2% of patients receiving sintilimab plus chemotherapy (Yang et al., 2020). Similarly, in the ORIENT-16 trial, the most common Grade ≥3 treatment-related adverse events in the sintilimab plus chemotherapy group were decreased platelet count (24.7%), decreased neutrophil count (20.1%), and anemia (12.5%) (Xu et al., 2023). This discrepancy highlights the limitations of clinical trial data, which often exclude patients with advanced disease, multiple comorbidities, or extensive prior treatment histories, thus underestimating the actual toxicity burden encountered in routine clinical practice. The FAERS analysis further substantiated myelosuppression as the most frequently reported hematological PT, with a strong disproportionality signal (ROR = 51.86), indicating a statistically significant association with sintilimab exposure. This finding supports the clinical relevance of myelosuppression as a safety concern, although it does not imply incidence. Collectively, these findings underscore the necessity of prioritizing myelosuppression monitoring in patients treated with sintilimab, particularly given its potential to lead to life-threatening complications such as severe infection or hemorrhage.

The identification of distinct risk factors for myelosuppression provides valuable insights for clinical risk stratification. Analysis of the FAERS dataset revealed that female sex (adjusted OR = 0.457, 95% CI: 0.245–0.847, p = 0.013) and concurrent administration of paclitaxel (adjusted OR = 4.129, 95% CI: 1.605–11.260, p = 0.004) are independent risk factors. The observed sex-related differences in myelosuppression risk align with previous findings in ICIs-related toxicity, suggesting that sex-specific variations in immune response—potentially influenced by hormonal differences or genetic polymorphisms—may affect susceptibility to irAEs (Pala and Conforti, 2021; Tao et al., 2025; Anobile et al., 2023).

Notably, the risk factors identified in the FAERS analysis differed in part from those observed in the clinical cohort. While FAERS highlighted female sex as protective and paclitaxel as a strong risk factor, the clinical cohort—composed predominantly of male patients with esophageal cancer—identified advanced M stage (OR = 0.871, 95% CI: 0.788–0.960) and cisplatin use (OR = 2.240, 95% CI: 1.133–4.450) as independent predictors. These discrepancies likely reflect fundamental differences in data source characteristics: FAERS captures global spontaneous reports with broader demographic and oncologic diversity but is susceptible to reporting bias, whereas the clinical cohort provides granular, longitudinal data from a homogeneous population with standardized follow-up. The protective effect of female sex observed in FAERS could not be evaluated in the clinical cohort due to the low proportion of female patients, underscoring the need for sex-balanced prospective studies. Conversely, the association with cisplatin in the clinical cohort—but not in FAERS—may reflect regional treatment practices or underreporting of specific chemotherapy combinations in spontaneous reports. Importantly, both datasets consistently identified concurrent chemotherapy as a key amplifier of myelosuppression risk, reinforcing the clinical message that sintilimab-based combination regimens warrant intensified hematologic monitoring. These complementary findings enhance the clinical relevance of our study by illustrating how real-world risk profiles may vary across different populations and care settings, and they support the development of context-specific risk stratification strategies.

The observation that female sex appears to confer a protective effect against myelosuppression, as indicated by the FAERS analysis, necessitates careful interpretation. Several factors may influence this finding. Firstly, the FAERS database is susceptible to reporting biases, such as the potential underreporting of hematological adverse events in female patients or the overrepresentation of male patients in specific cancer types associated with a higher risk of myelosuppression (Belančić et al., 2025; Xu et al., 2025; Yu et al., 2016). Secondly, the clinical cohort utilized in this study was predominantly male (97.45%), which may mirror real-world prescribing patterns in esophageal cancer. However, this demographic imbalance constrains the ability to substantiate sex-based differences in toxicity. Third, although certain studies indicate that females may experience irAEs more frequently but with less severity (Vitale et al., 2024), the association between sex and hematological toxicity remains inconclusive. Hypotheses regarding biological mechanisms, such as estrogen-mediated protection of hematopoietic stem cells or differences in drug metabolism, have been proposed but necessitate further investigation (Kumar and Goyal, 2021; Behal et al., 2025; Sakuma et al., 2009). Consequently, while our analysis of the FAERS identified female sex as a statistically protective factor, this finding should be interpreted with caution and does not suggest that female patients are exempt from the risk of myelosuppression. Future prospective studies with balanced sex representation are essential to elucidate the true impact of sex on sintilimab-related hematological toxicity.

Furthermore, paclitaxel is well-documented for its myelosuppressive effects, primarily affecting rapidly dividing hematopoietic progenitor cells (Guchelaar et al., 1994; Mitchison, 2012). Our findings indicate a synergistic toxic effect when paclitaxel is administered in conjunction with sintilimab, which may be attributed to ICIs-induced disruption of immune homeostasis, thereby exacerbating chemotherapy-induced bone marrow injury. Nevertheless, the specific biological interactions between sintilimab and these chemotherapeutic agents at the bone marrow level remain inadequately understood. Given the observational nature of this study, it lacks mechanistic insights into the modulation of chemotherapy-induced hematopoietic toxicity by PD-1 inhibition. Therefore, future laboratory investigations are imperative, including *in vitro* co-culture models of hematopoietic stem cells with immune effectors and *in vivo* murine studies, to determine whether this synergistic effect is mediated by T-cell overactivation, cytokine dysregulation, or direct impairment of bone marrow niche function. Additionally, such studies should investigate the potential involvement of myeloid-derived suppressor cells or inflammatory signaling pathways in exacerbating marrow suppression. Elucidating these mechanisms will be crucial for developing targeted preventive strategies and optimizing combination regimens.

Within the clinical cohort, advanced T/N/M tumor stages and concurrent use of cisplatin were associated with an increased risk of myelosuppression. The nephrotoxic and direct inhibitory effects of cisplatin on bone marrow function are well-documented (Chuang et al., 2022; Zhang et al., 2021), and the additive toxicity observed with sintilimab may result from impaired hematopoietic recovery in the context of immune activation. These risk factors collectively underscore the necessity for personalized pretreatment assessment, particularly for patients with advanced disease or those scheduled for combination chemotherapy.

TTO profile of adverse events associated with sintilimab offers essential insights for clinical surveillance. The median TTO of 14 days (interquartile range: 5–30 days) and the Weibull shape parameter (β = 0.785) suggest that the risk of adverse events, including myelosuppression, is most pronounced during the early phase of treatment and progressively decreases over time. This temporal pattern is consistent with the immunological mechanism of ICIs, wherein immune activation reaches its peak shortly after the initiation of therapy (Zhuang and Wu, 2023). Clinically, this underscores the necessity for rigorous hematological monitoring during the initial 4–6 weeks of sintilimab administration, with heightened attention during the first two treatment cycles. Based on the findings, it is recommended that complete blood counts be evaluated at baseline and subsequently monitored every one to 2 weeks during the initial 6 weeks of therapy. This is particularly advised for patients presenting with risk factors such as female sex, concurrent administration of paclitaxel or cisplatin, or advanced tumor stage. In populations deemed high-risk, more frequent monitoring, such as on a weekly basis, may be necessary to facilitate early detection and timely intervention. For patients at elevated risk, such as those undergoing treatment with paclitaxel or cisplatin or those with advanced tumor stages, more frequent assessments of blood counts may be required to facilitate early detection and prompt intervention, such as the administration of granulocyte colony-stimulating factor or adjustments in dosage.

In addition to myelosuppression, the pharmacovigilance analysis identified distinct patterns of adverse events across various SOCs. Endocrine disorders exhibited the most pronounced disproportionality signal, aligning with the well-documented ICIs-related endocrine irAEs such as hypothyroidism and hypophysitis (Yu et al., 2021; Trevisani et al., 2023). Remarkably, reactive capillary endothelial proliferation presented an exceptionally high ROR, a finding that may be attributed to the unique pharmacological characteristics of sintilimab or the underreporting of this event in other ICI datasets. Conversely, psychiatric, neurological, and musculoskeletal disorders exhibited negative signals, indicating a lower relative risk of these events with sintilimab compared to other medications in the FAERS database. This comprehensive safety profile underscores the necessity for multisystem monitoring in patients treated with sintilimab, rather than concentrating exclusively on hematological or endocrine toxicities.

This study is subject to several limitations that warrant acknowledgment. Firstly, the FAERS database is constrained by the inherent limitations associated with spontaneous reporting systems, including reporting bias—such as the overrepresentation of severe or unusual events—alongside incomplete data, exemplified by missing demographic or clinical details, and the possibility of duplicate reports. Furthermore, since sintilimab is primarily approved and used in China, the geographic distribution of reports in FAERS is predominantly from this region (97.31% in our dataset), which may limit the global generalizability of the pharmacovigilance findings. Additionally, the predominance of reports submitted by pharmacists and physicians (91.17%), with underrepresentation from consumers or patients, may reflect cultural, linguistic, or technical barriers to direct patient reporting, potentially skewing the detected safety signals. Another important consideration is that sintilimab is frequently administered in combination with chemotherapy or targeted agents; this polypharmacy context may confound the precise attribution of hematological toxicity, as myelosuppression could be exacerbated or primarily driven by concomitant medications rather than by sintilimab alone. The study’s single-center retrospective design may also cause selection bias, as the cohort is mostly male (97.65%) and primarily consists of esophageal cancer patients (70.00%), limiting generalizability. This gender imbalance accurately reflects the real-world demographics of esophageal cancer in our region but constrains the ability to substantiate sex-based comparisons and limits the generalizability of our findings to populations with a different demographic composition. Future multicenter prospective studies with balanced sex representation are needed to validate these findings. To address this, we used FAERS pharmacovigilance data, which offers a more diverse population. However, caution is needed when applying these findings broadly. Future multicenter prospective studies with balanced demographics are needed to confirm our results. Thirdly, the risk factor analysis did not consider potential confounding variables, such as baseline hematological parameters, comorbidities, prior radiotherapy, or cumulative drug doses, all of which may influence the development of myelosuppression. Moreover, the analysis of time-to-onset data obtained from the FAERS database may be complicated by unmeasured variables, including disease progression, concurrent infections, or alterations in clinical status, which are not accounted for in spontaneous reporting systems. Although our clinical cohort excluded patients with evident progression or active infection prior to the onset of the event, the potential for residual confounding persists. Consequently, the temporal patterns identified should be approached with caution, and future research incorporating longitudinal clinical data is necessary to substantiate these findings. Lastly, the absence of long-term follow-up data precludes the assessment of late-onset myelosuppression or recovery trajectories.

Despite these limitations, the integrative approach of combining pharmacovigilance signal mining with real-world clinical data enhances the validity of our findings, providing a more comprehensive understanding of sintilimab-associated myelosuppression than either method alone. Importantly, while this study identifies a high incidence of myelosuppression and associated risk factors, it does not explore the underlying biological mechanisms. Mechanistic studies—particularly those involving bone marrow samples—are essential to determine whether myelosuppression results from direct hematopoietic toxicity, immune-mediated destruction, or synergistic effects with concomitant chemotherapy. Future research should incorporate bone marrow examinations, along with immunological and hematological profiling, to clarify the pathophysiology of sintilimab-related hematotoxicity. The clinical implications of this study are significant: clinicians should perform pretreatment risk stratification based on sex, tumor stage, and planned concomitant medications; implement targeted hematological monitoring during the early treatment phase; and develop individualized management strategies for high-risk patients. Future research should aim to address the study’s limitations by conducting large-scale, multicenter prospective studies to validate the identified risk factors. Furthermore, mechanistic investigations are necessary to elucidate the molecular pathways underlying sintilimab-induced myelosuppression, particularly its synergistic effects with chemotherapy agents. The identification of predictive biomarkers, such as genetic polymorphisms, immune cell subsets, or serum cytokines, could further facilitate precision medicine approaches, enabling risk-adapted dosing, monitoring, and prevention.

The findings obtained from the FAERS database must be interpreted with caution due to several intrinsic limitations. Spontaneous reporting systems are prone to reporting biases, including the overrepresentation of severe or unusual events and the underreporting of common or anticipated toxicities. Additionally, the lack of comprehensive clinical data and the absence of a defined patient population denominator hinder the estimation of true incidence or causal attribution. Consequently, the disproportionality signals identified in this study should be considered as hypothesis-generating, with their clinical significance requiring evaluation through well-designed observational or prospective studies. To address this limitation, we integrated FAERS data with a real-world clinical cohort in this study, providing complementary evidence on the incidence, severity, and risk factors associated with myelosuppression.

## 5 Conclusion

This integrative analysis, combining pharmacovigilance signal detection with real-world clinical data, suggests that myelosuppression is a clinically significant adverse event associated with sintilimab therapy. While the FAERS data revealed a strong statistical signal for myelosuppression, the high incidence and severity were substantiated by the clinical cohort. These findings underscore the need for vigilant monitoring and risk stratification in clinical practice. The analysis identifies key risk factors, such as female sex, concomitant administration of paclitaxel or cisplatin, and advanced tumor stage, which predispose patients to this hematological toxicity. The time-to-onset profile, characterized by the highest risk occurring in the early phase of treatment, highlights the critical importance of rigorous hematological monitoring during the initial weeks of therapy. These findings underscore the necessity for personalized risk stratification and proactive management strategies to optimize the safe administration of sintilimab in clinical settings. Future prospective studies and mechanistic investigations are essential to validate these risk factors and elucidate the underlying biological mechanisms.

## Data Availability

The original contributions presented in the study are included in the article/Supplementary Material, further inquiries can be directed to the corresponding authors.
