# Supplementary material for: A multidimensional investigation of myelosuppression associated with sintilimab: integrating pharmacovigilance signal mining with real-world clinical evidence
Source: Front Pharmacol. 2026 Apr 10;17:1784033. doi: 10.3389/fphar.2026.1784033 (PMC13106480; doi:10.3389/fphar.2026.1784033)
Supplement: Supplementary file 1 [file Table1.docx]

**Table S1** Two-by-Two Table for Detecting Signals.

|  | Drug-related AEs | Non-drug-related AEs | Total |
| --- | --- | --- | --- |
| Drug | $\text{a}$ | $\text{b}$ | $\text{a}\text{+}\text{b}$ |
| Non-drug | $\text{c}$ | $\text{d}$ | $\text{c}\text{+}\text{d}$ |
| Total | $\text{a}\text{+}\text{c}$ | $\text{b}\text{+}\text{d}$ | $\text{N}\text{=}\text{a}\text{+}\text{b}\text{+}\text{c}\text{+}\text{d}$ |
