# Supplementary material for: A multidimensional investigation of myelosuppression associated with sintilimab: integrating pharmacovigilance signal mining with real-world clinical evidence
Source: Front Pharmacol. 2026 Apr 10;17:1784033. doi: 10.3389/fphar.2026.1784033 (PMC13106480; doi:10.3389/fphar.2026.1784033)
Supplement: Supplementary file 2 [file Table2.docx]

**Table S2** This research employs ROR, PRR, IC and EBGM, along with their respective formulas and thresholds.

| Algorithms | Calculation formula | Criteria |
| --- | --- | --- |
| ROR | $ROR=\frac{a/c}{b/d}=\frac{ad}{bc}$  $95\%CI=e^{In(ROR)\pm1.96\sqrt{\frac{1}{a}+\frac{1}{b}+\frac{1}{c}+\frac{1}{d}}}$ | $a\geq3$  $ROR\geq2$  $95\%CI>1$ |
| PRR | $PRR=\frac{a/(a+b)}{c/(c+d)}=\frac{a(c+d)}{c(a+b)}$  $\chi^{2}=\frac{\left( \left\vert ad-bc \right\vert-\frac{n}{2} \right)^{2}n}{(a+b)(a+c)(c+d)(b+d)}$  $n=a+b+c+d$ | $a\geq3$  $PRR\geq2$  $\chi^{2}\geq4$ |
| BCPNN | $E\left( IC \right)={log}_{2}\frac{\left( C_{xy}+\gamma_{11} \right)\left( C+\alpha\right)\left( C+\beta\right)}{\left( C+\gamma\right)\left( C_{x}+\alpha_{1} \right)\left( C_{y}+\beta_{1} \right)}$  $V\left( IC \right)=\frac{1}{{(In2)}^{2}}\left\{ \left[ \frac{C-C_{xy}+\gamma-\gamma_{11}}{\left( C_{xy}+\gamma_{11} \right)\left( 1+C+\gamma\right)} \right]+\left[ \frac{C-C_{x}+\alpha-\alpha_{1}}{\left( C_{x}+\alpha_{1} \right)\left( 1+C+\alpha\right)} \right]+\left[ \frac{C-C_{x}+\alpha-\alpha_{1}}{\left( C_{y}+\beta_{1} \right)\left( 1+C+\beta\right)} \right] \right\}$  $\gamma=\gamma_{11}\frac{(C+\alpha)(C+\beta)}{(C_{x}+\alpha_{1})(C_{y}+\beta_{1})}$  $IC-2SD=E\left( IC \right)-2\sqrt{V(IC)}$  $\alpha_{1}=\beta_{1}=1$  $\alpha=\beta=2$  $\gamma_{11}=1$  $C=a+b+c+d$  $C_{x}=a+b$  $C_{y}=a+c$  $C_{xy}=a$ | $a\geq3$  $IC-2SD>0$ |
| EBGM^*^ | $EBGM=a(a+b+c+d)/[(a+c)(a+b)]$ | $a>0$  $95\%CI>2$ |

*MGPS/EBGM employs Bayesian methods to compute an empirical Bayes geometric mean (EBGM) estimate of the relative reporting strength for a specific drug-adverse event pair and its uncertainty. This estimate integrates the overall reporting pattern within the database as a dynamic prior (baseline) with the observed report count (likelihood). The method is particularly effective for analyzing sparse data from rare events, utilizing contraction estimation to adjust estimates toward the overall mean.

IC: Information Component
