# Supplementary material for: A multidimensional investigation of myelosuppression associated with sintilimab: integrating pharmacovigilance signal mining with real-world clinical evidence
Source: Front Pharmacol. 2026 Apr 10;17:1784033. doi: 10.3389/fphar.2026.1784033 (PMC13106480; doi:10.3389/fphar.2026.1784033)
Supplement: Supplementary file 3 [file Table5.docx]

**Table S5** Outcomes of hematological examinations in clinical practice for patients before and after Sintilimab administration.

| Variables | before (n = 170) | after (n = 170) | p |
| --- | --- | --- | --- |
| White Blood Cell Count, Median (Q1,Q3) | 5.49 (4.5, 7.58) | 4.88 (3.96, 6.54) | 0.002 |
| Red Blood Cell Count, Mean ± SD | 3.95 ± 0.58 | 3.83 ± 0.62 | 0.066 |
| Hemoglobin, Mean ± SD | 120.66 ± 17.87 | 117.2 ± 18.77 | 0.082 |
| Hematocrit, Mean ± SD | 36.84 ± 5.17 | 35.52 ± 5.5 | 0.023 |
| Platelet Count, Median (Q1,Q3) | 151 (94.85, 228.25) | 138.5 (93.1, 210.5) | 0.25 |
| Neutrophil Percentage, Median (Q1,Q3) | 60.4 (31.8, 69.92) | 60.9 (31.22, 73.27) | 0.765 |
| Lymphocyte Percentage, Median (Q1,Q3) | 29.4 (20.42, 323.75) | 32.1 (19.23, 324.75) | 0.694 |
| Monocyte Percentage, Median (Q1,Q3) | 8.6 (5.82, 168) | 8.1 (3.42, 142.5) | 0.022 |
| Eosinophil Percentage, Median (Q1,Q3) | 2.55 (1, 62.13) | 2.25 (0.7, 63.22) | 0.632 |
| Basophil Percentage, Median (Q1,Q3) | 0.6 (0.3, 18.6) | 0.6 (0.2, 16.62) | 0.587 |
| Neutrophil Absolute Count, Median (Q1,Q3) | 5.28 (3.07, 7.8) | 3.38 (2.3, 5.34) | < 0.001 |
| Lymphocyte Absolute Count, Median (Q1,Q3) | 1.36 (0.94, 1.86) | 1.12 (0.69, 1.65) | 0.004 |
| Monocyte Absolute Count, Median (Q1,Q3) | 0.4 (0.25, 0.6) | 0.3 (0.15, 0.46) | < 0.001 |
| Eosinophil Absolute Count, Median (Q1,Q3) | 0.16 (0.06, 3.07) | 0.11 (0.04, 2.56) | 0.127 |
| Basophil Absolute Count, Median (Q1,Q3) | 0.03 (0.02, 0.93) | 0.03 (0.01, 0.86) | 0.105 |
| Mean Corpuscular Volume, Median (Q1,Q3) | 89.65 (0.65, 94.8) | 89.45 (0.29, 94.1) | 0.05 |
| Mean Corpuscular Hemoglobin, Median (Q1,Q3) | 29.1 (0.17, 31.17) | 29.35 (0.12, 31.17) | 0.574 |
| Mean Corpuscular Hemoglobin Concentration, Median (Q1,Q3) | 319 (0.05, 329) | 322 (0.03, 331) | 0.734 |
| Red Blood Cell Distribution Width SD, Median (Q1,Q3) | 47.8 (43.9, 53.48) | 47.8 (43.9, 53.08) | 0.788 |
| Red Blood Cell Distribution Width CV, Median (Q1,Q3) | 14.15 (12.8, 15.67) | 14.4 (12.9, 15.6) | 0.808 |
| Plateletcrit, Median (Q1,Q3) | 0.19 (0.15, 0.24) | 0.17 (0.14, 0.22) | 0.035 |
| Mean Platelet Volume, Median (Q1,Q3) | 9.25 (8.5, 9.9) | 9.6 (8.6, 10.4) | 0.043 |
| Platelet Distribution Width, Median (Q1,Q3) | 15.8 (11.12, 16.2) | 15.9 (12.3, 16.28) | 0.122 |
| Large Platelet Ratio, Median (Q1,Q3) | 20.3 (15.6, 24.65) | 22.85 (16.3, 28.17) | 0.011 |
| Grades of myelosuppression, n (%) |  |  | 0.03 |
| 0 | 65 (38.24) | 56 (32.94) |  |
| 1 | 86 (50.59) | 69 (40.59) |  |
| 2 | 16 (9.41) | 32 (18.82) |  |
| 3 | 3 (1.76) | 7 (4.12) |  |
| 4 | 0 (0) | 6 (3.53) |  |
