# Supplementary material for: A multidimensional investigation of myelosuppression associated with sintilimab: integrating pharmacovigilance signal mining with real-world clinical evidence
Source: Front Pharmacol. 2026 Apr 10;17:1784033. doi: 10.3389/fphar.2026.1784033 (PMC13106480; doi:10.3389/fphar.2026.1784033)
Supplement: Supplementary file 4 [file Table3.docx]

**Table S3** Adverse events associated with Sintilimab and their PTs characteristics.

| **SOC** | **PT** | **Case Reports** | **ROR(95% CI)** | **PRR(95% CI)** | **IC**  **(IC025)** | **EBGM**  **(EBGM05)** |
| --- | --- | --- | --- | --- | --- | --- |
| Blood and lymphatic system disorders | Myelosuppression | 146 | 51.86(43.88, 61.28) | 49.25(42.1, 57.61) | 5.61(5.37) | 48.97(42.59) |
| Skin and subcutaneous tissue disorders | Rash | 67 | 3.26(2.56, 4.15) | 3.21(2.54, 4.06) | 1.68(1.33) | 3.21(2.62) |
| Gastrointestinal disorders | Vomiting | 60 | 3.16(2.45, 4.08) | 3.12(2.42, 4.03) | 1.64(1.27) | 3.12(2.52) |
| Endocrine disorders | Hypothyroidism | 51 | 32.87(24.91, 43.39) | 32.3(24.55, 42.5) | 5.01(4.61) | 32.18(25.52) |
| Blood and lymphatic system disorders | Anaemia | 51 | 6.68(5.06, 8.81) | 6.57(4.99, 8.64) | 2.72(2.32) | 6.57(5.21) |
| Investigations | Platelet count decreased | 50 | 9.87(7.46, 13.05) | 9.71(7.38, 12.78) | 3.28(2.88) | 9.7(7.68) |
| General disorders and administration site conditions | Therapy partial responder | 45 | 36.75(27.36, 49.36) | 36.18(26.96, 48.55) | 5.17(4.75) | 36.03(28.15) |
| Investigations | White blood cell count decreased | 44 | 8.13(6.04, 10.96) | 8.02(5.98, 10.76) | 3(2.58) | 8.02(6.25) |
| Metabolism and nutrition disorders | Decreased appetite | 39 | 3.61(2.63, 4.95) | 3.57(2.61, 4.88) | 1.84(1.39) | 3.57(2.74) |
| Skin and subcutaneous tissue disorders | Palmar-plantar erythrodysaesthesia syndrome | 35 | 31.98(22.9, 44.67) | 31.6(22.65, 44.1) | 4.98(4.5) | 31.49(23.81) |
| Blood and lymphatic system disorders | Thrombocytopenia | 35 | 7.08(5.07, 9.88) | 7.01(5.02, 9.78) | 2.81(2.33) | 7(5.3) |
| Vascular disorders | Hypertension | 35 | 3.76(2.69, 5.24) | 3.72(2.67, 5.19) | 1.9(1.42) | 3.72(2.81) |
| Blood and lymphatic system disorders | Neutropenia | 35 | 4.52(3.24, 6.31) | 4.48(3.21, 6.25) | 2.16(1.69) | 4.48(3.39) |
| Investigations | Alanine aminotransferase increased | 33 | 14.78(10.48, 20.84) | 14.62(10.48, 20.4) | 3.87(3.38) | 14.6(10.95) |
| General disorders and administration site conditions | Disease progression | 33 | 5.48(3.89, 7.72) | 5.43(3.89, 7.58) | 2.44(1.95) | 5.42(4.07) |
| Investigations | Aspartate aminotransferase increased | 32 | 17.27(12.19, 24.48) | 17.09(12.01, 24.32) | 4.09(3.6) | 17.06(12.74) |
| Hepatobiliary disorders | Hepatic function abnormal | 31 | 18.21(12.77, 25.95) | 18.02(12.66, 25.64) | 4.17(3.67) | 17.98(13.37) |
| Blood and lymphatic system disorders | Leukopenia | 31 | 14.24(9.99, 20.29) | 14.1(9.91, 20.06) | 3.82(3.31) | 14.07(10.46) |
| Investigations | Neutrophil count decreased | 26 | 11.77(8, 17.33) | 11.68(7.89, 17.29) | 3.54(3) | 11.66(8.44) |
| Cardiac disorders | Myocarditis | 22 | 32.92(21.62, 50.11) | 32.67(21.65, 49.31) | 5.02(4.43) | 32.55(22.9) |
| Renal and urinary disorders | Proteinuria | 19 | 20.64(13.14, 32.42) | 20.51(13.07, 32.19) | 4.35(3.72) | 20.46(14.02) |
| Endocrine disorders | Hyperthyroidism | 18 | 27.73(17.43, 44.11) | 27.56(17.22, 44.11) | 4.78(4.13) | 27.47(18.63) |
| Investigations | Blood bilirubin increased | 17 | 18.83(11.68, 30.35) | 18.73(11.7, 29.98) | 4.22(3.55) | 18.69(12.53) |
| Respiratory, thoracic and mediastinal disorders | Pneumonitis | 15 | 10.68(6.43, 17.75) | 10.63(6.39, 17.69) | 3.41(2.7) | 10.62(6.94) |
| Hepatobiliary disorders | Liver injury | 14 | 9.75(5.77, 16.5) | 9.71(5.72, 16.48) | 3.28(2.55) | 9.7(6.25) |
| Metabolism and nutrition disorders | Hypoalbuminaemia | 14 | 41.31(24.4, 69.93) | 41.11(24.22, 69.79) | 5.35(4.62) | 40.91(26.34) |
| Cardiac disorders | Immune-mediated myocarditis | 13 | 94.15(54.44, 162.84) | 93.73(54.14, 162.26) | 6.53(5.77) | 92.7(58.61) |
| Gastrointestinal disorders | Gastrointestinal haemorrhage | 13 | 4.96(2.87, 8.55) | 4.94(2.85, 8.55) | 2.3(1.55) | 4.94(3.13) |
| Hepatobiliary disorders | Drug-induced liver injury | 13 | 7.07(4.1, 12.19) | 7.04(4.07, 12.19) | 2.81(2.06) | 7.04(4.46) |
| Hepatobiliary disorders | Hepatitis | 12 | 11.21(6.36, 19.77) | 11.17(6.33, 19.72) | 3.48(2.69) | 11.15(6.94) |
| Hepatobiliary disorders | Hepatic failure | 11 | 10.59(5.86, 19.15) | 10.55(5.86, 18.99) | 3.4(2.58) | 10.54(6.42) |
| Investigations | Granulocyte count decreased | 11 | 99.11(54.63, 179.79) | 98.73(54.84, 177.75) | 6.61(5.79) | 97.59(59.29) |
| Hepatobiliary disorders | Hyperbilirubinaemia | 11 | 25.26(13.96, 45.71) | 25.17(13.98, 45.32) | 4.65(3.83) | 25.1(15.28) |
| Investigations | Transaminases increased | 11 | 11.61(6.42, 21.01) | 11.57(6.43, 20.83) | 3.53(2.71) | 11.56(7.04) |
| Gastrointestinal disorders | Mouth ulceration | 10 | 10.42(5.6, 19.4) | 10.39(5.55, 19.45) | 3.38(2.52) | 10.38(6.17) |
| Investigations | Blood creatinine increased | 10 | 3.79(2.04, 7.05) | 3.78(2.02, 7.08) | 1.92(1.06) | 3.78(2.25) |
| General disorders and administration site conditions | Drug resistance | 9 | 6.28(3.26, 12.09) | 6.27(3.28, 11.97) | 2.65(1.75) | 6.26(3.62) |
| Gastrointestinal disorders | Ascites | 9 | 7.69(4, 14.81) | 7.67(4.02, 14.65) | 2.94(2.04) | 7.67(4.43) |
| Metabolism and nutrition disorders | Hypocalcaemia | 8 | 9.81(4.9, 19.65) | 9.79(4.93, 19.44) | 3.29(2.34) | 9.78(5.47) |
| Investigations | Interleukin level increased | 8 | 215.79(106.86, 435.74) | 215.18(106.26, 435.75) | 7.71(6.76) | 209.83(116.54) |
| Nervous system disorders | Neurotoxicity | 8 | 8.18(4.09, 16.38) | 8.16(4.11, 16.2) | 3.03(2.08) | 8.15(4.56) |
| Gastrointestinal disorders | Colitis | 8 | 4.07(2.03, 8.15) | 4.06(2.04, 8.06) | 2.02(1.08) | 4.06(2.27) |
| Endocrine disorders | Adrenal insufficiency | 8 | 11.05(5.52, 22.13) | 11.02(5.55, 21.88) | 3.46(2.52) | 11.01(6.16) |
| Respiratory, thoracic and mediastinal disorders | Bronchopleural fistula | 8 | 391.43(192.47, 796.04) | 390.33(192.75, 790.44) | 8.54(7.58) | 373.03(205.96) |
| Skin and subcutaneous tissue disorders | Dermatitis | 7 | 6.82(3.25, 14.33) | 6.81(3.23, 14.34) | 2.77(1.76) | 6.8(3.66) |
| Nervous system disorders | Myasthenia gravis | 7 | 13.07(6.22, 27.45) | 13.04(6.19, 27.46) | 3.7(2.7) | 13.02(7) |
| Hepatobiliary disorders | Immune-mediated hepatitis | 7 | 42.46(20.18, 89.3) | 42.35(20.11, 89.19) | 5.4(4.39) | 42.15(22.62) |
| Skin and subcutaneous tissue disorders | Immune-mediated dermatitis | 7 | 91.16(43.24, 192.16) | 90.94(43.18, 191.52) | 6.49(5.48) | 89.97(48.21) |
| Skin and subcutaneous tissue disorders | Toxic epidermal necrolysis | 7 | 11.15(5.31, 23.42) | 11.12(5.28, 23.42) | 3.47(2.47) | 11.11(5.97) |
| Respiratory, thoracic and mediastinal disorders | Haemoptysis | 7 | 6.41(3.05, 13.46) | 6.4(3.04, 13.48) | 2.68(1.67) | 6.39(3.44) |
| Investigations | Lymphocyte count decreased | 7 | 7.12(3.39, 14.95) | 7.1(3.37, 14.95) | 2.83(1.83) | 7.1(3.82) |
| Immune system disorders | Reactive capillary endothelial proliferation | 7 | 1549.72(691.48, 3473.19) | 1545.92(692.13, 3452.92) | 10.35(9.27) | 1305.6(664.59) |
| Investigations | Gamma-glutamyltransferase increased | 6 | 8.81(3.95, 19.63) | 8.79(3.94, 19.63) | 3.13(2.06) | 8.78(4.49) |
| Musculoskeletal and connective tissue disorders | Myositis | 6 | 14.48(6.49, 32.27) | 14.45(6.47, 32.28) | 3.85(2.78) | 14.43(7.38) |
| Blood and lymphatic system disorders | Haematotoxicity | 6 | 12.48(5.6, 27.83) | 12.46(5.58, 27.83) | 3.64(2.57) | 12.44(6.36) |
| Endocrine disorders | Thyroid disorder | 6 | 8.32(3.73, 18.55) | 8.31(3.72, 18.56) | 3.05(1.98) | 8.3(4.25) |
| Skin and subcutaneous tissue disorders | Drug eruption | 6 | 7.4(3.32, 16.48) | 7.38(3.3, 16.48) | 2.88(1.81) | 7.38(3.77) |
| Endocrine disorders | Hypophysitis | 6 | 56.06(25.1, 125.23) | 55.95(25.05, 124.97) | 5.8(4.72) | 55.58(28.37) |
| Cardiac disorders | Pericardial effusion | 5 | 5.07(2.11, 12.2) | 5.06(2.09, 12.22) | 2.34(1.18) | 5.06(2.43) |
| Neoplasms benign, malignant and unspecified (incl cysts and polyps) | Hepatocellular carcinoma | 5 | 17.7(7.35, 42.59) | 17.67(7.31, 42.69) | 4.14(2.98) | 17.63(8.46) |
| Cardiac disorders | Cardiotoxicity | 5 | 9.71(4.04, 23.35) | 9.69(4.01, 23.41) | 3.28(2.12) | 9.68(4.65) |
| Immune system disorders | Haemophagocytic lymphohistiocytosis | 5 | 8.14(3.38, 19.58) | 8.13(3.37, 19.64) | 3.02(1.87) | 8.12(3.9) |
| General disorders and administration site conditions | Hyperpyrexia | 5 | 24.68(10.25, 59.42) | 24.64(10.2, 59.52) | 4.62(3.46) | 24.57(11.78) |
| Gastrointestinal disorders | Immune-mediated enterocolitis | 5 | 15.87(6.6, 38.2) | 15.85(6.56, 38.29) | 3.98(2.83) | 15.82(7.59) |
| Gastrointestinal disorders | Gingival bleeding | 5 | 8.24(3.43, 19.82) | 8.23(3.41, 19.88) | 3.04(1.88) | 8.22(3.94) |
| Cardiac disorders | Acute myocardial infarction | 5 | 5.73(2.38, 13.78) | 5.72(2.37, 13.82) | 2.52(1.36) | 5.72(2.74) |
| Investigations | Blood albumin decreased | 5 | 19.38(8.05, 46.64) | 19.35(8.01, 46.74) | 4.27(3.11) | 19.3(9.26) |
| Investigations | Amylase increased | 5 | 30.33(12.59, 73.03) | 30.27(12.53, 73.12) | 4.92(3.76) | 30.17(14.46) |
| Respiratory, thoracic and mediastinal disorders | Immune-mediated lung disease | 5 | 33.12(13.75, 79.78) | 33.07(13.69, 79.89) | 5.04(3.88) | 32.94(15.79) |
| Renal and urinary disorders | Nephritis | 5 | 31.39(13.03, 75.6) | 31.34(12.97, 75.71) | 4.96(3.81) | 31.22(14.97) |
| Gastrointestinal disorders | Upper gastrointestinal haemorrhage | 5 | 7.08(2.94, 17.04) | 7.07(2.93, 17.08) | 2.82(1.66) | 7.07(3.39) |
| Blood and lymphatic system disorders | Agranulocytosis | 4 | 4.69(1.76, 12.51) | 4.68(1.76, 12.47) | 2.23(0.96) | 4.68(2.06) |
| Skin and subcutaneous tissue disorders | Skin toxicity | 4 | 13.25(4.97, 35.37) | 13.24(4.97, 35.28) | 3.72(2.46) | 13.22(5.81) |
| Neoplasms benign, malignant and unspecified (incl cysts and polyps) | Metastases to lymph nodes | 4 | 11.89(4.46, 31.73) | 11.88(4.46, 31.65) | 3.57(2.3) | 11.86(5.22) |
| Respiratory, thoracic and mediastinal disorders | Atelectasis | 4 | 12.67(4.75, 33.81) | 12.65(4.75, 33.71) | 3.66(2.39) | 12.64(5.56) |
| Infections and infestations | Pneumonia bacterial | 4 | 7.19(2.7, 19.19) | 7.19(2.7, 19.16) | 2.84(1.58) | 7.18(3.16) |
| Infections and infestations | Pneumonia fungal | 4 | 15.88(5.95, 42.37) | 15.86(5.95, 42.26) | 3.98(2.72) | 15.83(6.96) |
| Investigations | Blood alkaline phosphatase increased | 4 | 5.73(2.15, 15.29) | 5.73(2.15, 15.27) | 2.52(1.25) | 5.72(2.52) |
| Investigations | Blood lactate dehydrogenase increased | 4 | 7.96(2.98, 21.24) | 7.95(2.98, 21.18) | 2.99(1.72) | 7.95(3.5) |
| Metabolism and nutrition disorders | Hypertriglyceridaemia | 4 | 14.95(5.6, 39.9) | 14.93(5.6, 39.78) | 3.9(2.63) | 14.91(6.56) |
| Respiratory, thoracic and mediastinal disorders | Nasal necrosis | 4 | 730.78(262.89, 2031.41) | 729.75(263.35, 2022.12) | 9.39(8.06) | 671.45(285.43) |
| Metabolism and nutrition disorders | Type 1 diabetes mellitus | 4 | 14.25(5.34, 38.04) | 14.24(5.34, 37.94) | 3.83(2.56) | 14.21(6.25) |
| Investigations | Lipase increased | 4 | 14.89(5.58, 39.72) | 14.87(5.58, 39.62) | 3.89(2.62) | 14.84(6.53) |
| Cardiac disorders | Myocardial injury | 4 | 49.73(18.6, 132.97) | 49.66(18.64, 132.32) | 5.63(4.36) | 49.37(21.68) |
| Renal and urinary disorders | Nephropathy toxic | 4 | 7.32(2.74, 19.51) | 7.31(2.74, 19.48) | 2.87(1.6) | 7.3(3.21) |
| Metabolism and nutrition disorders | Hypoproteinaemia | 4 | 50.85(19.02, 135.99) | 50.78(19.06, 135.3) | 5.66(4.39) | 50.49(22.17) |
| Gastrointestinal disorders | Gastrointestinal toxicity | 4 | 16.07(6.02, 42.9) | 16.05(6.02, 42.76) | 4(2.74) | 16.03(7.05) |
| Infections and infestations | Abdominal infection | 4 | 31.24(11.7, 83.45) | 31.2(11.71, 83.13) | 4.96(3.69) | 31.09(13.66) |
| Blood and lymphatic system disorders | Bone marrow failure | 3 | 6.29(2.03, 19.54) | 6.29(2.02, 19.6) | 2.65(1.24) | 6.29(2.44) |
| Congenital, familial and genetic disorders | Epidermolysis bullosa | 3 | 200.02(63.61, 628.93) | 199.81(64.11, 622.76) | 7.61(6.17) | 195.19(74.84) |
| Neoplasms benign, malignant and unspecified (incl cysts and polyps) | Metastasis | 3 | 8.72(2.81, 27.06) | 8.71(2.79, 27.15) | 3.12(1.71) | 8.7(3.37) |
| Congenital, familial and genetic disorders | Gene mutation | 3 | 22.46(7.23, 69.79) | 22.44(7.2, 69.94) | 4.48(3.07) | 22.38(8.67) |
| Metabolism and nutrition disorders | Electrolyte imbalance | 3 | 5.66(1.82, 17.58) | 5.66(1.82, 17.64) | 2.5(1.08) | 5.66(2.19) |
| Metabolism and nutrition disorders | Hypophosphataemia | 3 | 8.45(2.72, 26.24) | 8.45(2.71, 26.34) | 3.08(1.66) | 8.44(3.27) |
| Blood and lymphatic system disorders | Hypoglobulinaemia | 3 | 475.53(148.51, 1522.63) | 475.03(149.45, 1509.87) | 8.81(7.35) | 449.63(169.81) |
| General disorders and administration site conditions | Temperature intolerance | 3 | 6.03(1.94, 18.71) | 6.02(1.93, 18.76) | 2.59(1.17) | 6.02(2.33) |
| Nervous system disorders | Peripheral sensory neuropathy | 3 | 11.26(3.63, 34.96) | 11.25(3.61, 35.06) | 3.49(2.07) | 11.24(4.35) |
| Respiratory, thoracic and mediastinal disorders | Pharyngeal necrosis | 3 | 969.34(293.23, 3204.44) | 968.33(292.94, 3200.85) | 9.76(8.26) | 868.26(319.27) |
| Renal and urinary disorders | Immune-mediated nephritis | 3 | 81.56(26.15, 254.43) | 81.48(26.14, 253.96) | 6.33(4.91) | 80.7(31.15) |
| Hepatobiliary disorders | Hypertransaminasaemia | 3 | 6.14(1.98, 19.07) | 6.14(1.97, 19.14) | 2.62(1.2) | 6.13(2.38) |
| Investigations | Blood thyroid stimulating hormone increased | 3 | 8.83(2.84, 27.4) | 8.82(2.83, 27.49) | 3.14(1.72) | 8.81(3.41) |
| Endocrine disorders | Thyroiditis | 3 | 17.49(5.63, 54.32) | 17.47(5.61, 54.45) | 4.12(2.71) | 17.44(6.75) |
| Gastrointestinal disorders | Chronic gastritis | 3 | 21.88(7.04, 67.97) | 21.85(7.01, 68.1) | 4.45(3.03) | 21.8(8.44) |
| Gastrointestinal disorders | Oesophagitis | 3 | 7.52(2.42, 23.36) | 7.52(2.41, 23.44) | 2.91(1.49) | 7.51(2.91) |
