# Supplementary material for: A multidimensional investigation of myelosuppression associated with sintilimab: integrating pharmacovigilance signal mining with real-world clinical evidence
Source: Front Pharmacol. 2026 Apr 10;17:1784033. doi: 10.3389/fphar.2026.1784033 (PMC13106480; doi:10.3389/fphar.2026.1784033)
Supplement: Supplementary file 5 [file Table4.docx]

**Table S4** The features of the clinical treatment practices for patients.

| **Variables** | **Patients (n = 170)** |
| --- | --- |
| **Patient characteristics** |  |
| Age(years), Mean±SD | 63.51±8.63 |
| Sex, n(%) |  |
| Male | 166 (97.65%) |
| Female | 4 (2.35%) |
| **Stage** |  |
| T, n (%) |  |
| T1 | 8 (4.71%) |
| T2 | 19 (11.18%) |
| T3 | 69 (40.59%) |
| T4 | 41 (24.12%) |
| Unknown | 33 (19.40%) |
| N, n (%) |  |
| N0 | 10 (5.88%) |
| N1 | 49 (28.82%) |
| N2 | 47 (27.65%) |
| N3 | 36 (21.18%) |
| Unknown | 28 (16.47%) |
| M, n (%) |  |
| M0 | 90 (52.94%) |
| M1 | 51 (30.00%) |
| Unknown | 29 (17.06%) |
| Stage, n (%) |  |
| Ⅰ | 1 (0.59%) |
| Ⅱ | 22 (12.94%) |
| Ⅲ | 58 (34.12%) |
| Ⅳ | 89 (52.35%) |
| **Cancer** |  |
| Lung cancer, n (%) |  |
| No | 119 (70.00%) |
| Yes | 51 (30.00%) |
| Esophageal cancer, n (%) |  |
| No | 51 (30.00%) |
| Yes | 119 (70.00%) |
| **Drug** |  |
| Carboplatin, n (%) |  |
| No | 143 (84.12%) |
| Yes | 27 (15.88%) |
| Nedaplatin, n (%) |  |
| No | 147 (86.47%) |
| Yes | 23 (13.53%) |
| Cisplatin, n (%) |  |
| No | 65 (38.24%) |
| Yes | 105 (61.76%) |
| Bevacizumab, n (%) |  |
| No | 164 (96.47%) |
| Yes | 6 (3.53%) |
